# Early post-hatching effects of antibiotics and ionophore coccidiostat supplementation on immune parameters in turkeys

**DOI:** 10.1016/j.psj.2025.105782

**Published:** 2025-09-04

**Authors:** B. Tykałowski, J. Kowalczyk, K. Ognik, D. Mikulski, A. Koncicki, K. Kozłowski, J. Jankowski

**Affiliations:** a Department of Poultry Diseases, Faculty of Veterinary Medicine, University of Warmia and Mazury in Olsztyn, 10-719 Olsztyn, Poland; b Department of Biochemistry and Toxicology, Faculty of Animal Sciences and Bioeconomy, University of Life Sciences, 20-950 Lublin, Poland; c Department of Poultry Science and Apiculture, Faculty of Animal Bioengineering, University of Warmia and Mazury in Olsztyn, 10-719 Olsztyn, Poland

**Keywords:** Enrofloxacin, Doxycycline, Monensin, Immunomodulation, Turkeys

## Abstract

Numerous factors can disrupt the development and function of the turkey immune system. The post-hatching period is critical because poults are exposed to various substances, microorganisms, and vaccines when their immune system is immature. The aim of this experiment was to determine whether the administration of antimicrobials to young turkeys affects their immune responses. The experiment was designed two-factorially, with four treatments – C (untreated control), M, E, and D –and two vaccination schedules – vaccinated (+) and unvaccinated (−). Groups *M*+ and *M*− received the coccidiostat monensin in their diet for 56 days. Groups *E*+ and *E*− were administered enrofloxacin, and those from groups *D*+ and *D*− doxycycline in drinking water for the first 5 days of life. One-day-old turkeys from groups *C*+, *M*+, *E*+, and *D*+ were vaccinated against turkey rhinotracheitis and Newcastle disease, and 28-day-old turkeys against *Ornithobacterium rhinotracheale*. On days 7 and 56, the levels and relative gene expression of selected cytokines, and the percentages of T cells (CD4^+^, CD8a^+^, and CD4^+^CD8a^+^) and B cells (IgM^+^) were determined in blood samples, as were the levels of these cells in the spleen. The coccidiostat had no effect on the percentage of the analyzed T- and B-cell subpopulations, but it increased the plasma IL-8 and interferon γ levels in 7-day-old birds. Antibiotics decreased the plasma IL-6 and tumor necrosis factor α levels in 7- and 56-day-old turkeys. Doxycycline increased the splenic percentages of CD8^+^ and CD4^+^CD8^+^*T* cells in 56-day-old birds. Enrofloxacin decreased the percentage of B cells and IL-6 gene expression in blood and the plasma levels of IL-1β and IL-2 in the 7-day-old birds. The antibiotics administered to turkeys interfered with the vaccine–immune system interaction by modulating the vaccine-induced response and exerting anti-inflammatory effects. Therefore, administration of antibiotics to healthy poults may suppress immunity, reduce vaccine-induced immunity, and increase susceptibility to infection.

## Introduction

The immune response in birds at different stages of rearing is determined by the maturation of the immune system, health status, and body condition. When birds are exposed to numerous infectious and non-infectious agents, among which are a wide variety of environmental factors, those agents can disrupt the development of the immune system both during incubation and in the first days after hatching. This makes the post-hatching period critical for poults from an immunological point of view. The immune system of newly hatched turkey poults is not fully mature and therefore may not provide complete protection against pathogens upon the birds’ first contact with the environment. Innate immune mechanisms appear to be quite functional in poults, but optimal adaptive immune responses are not developed until the first few weeks after hatching (Härtle et al., 2022). During this period, the development of innate defenses is primarily stimulated by interaction with commensal microbiota (Jiao et al., 2009; Thaiss et al., 2016; Kogut et al., 2020). The gut microbiome plays an important role in immune activation; therefore, disruptions in early life microbial colonization may affect host immunity later in life (Simon, 2016). Developing turkey embryos and poults are also protected against pathogens by maternal antibodies transferred via the egg yolk (Murai, 2013; Kowalczyk et al., 2019; Murai et al., 2020). The transfer of maternal antibodies helps protect poults until the adaptive immune response becomes fully effective. The administration of antibiotics immediately after hatching severely disrupts the composition of the gut microbiome (Shen et al., 2024). It may cause the loss of specific microbial populations and thereby dysregulate the production of antimicrobial peptides or metabolites against pathogen colonization. Disruption of the intestinal microbiota by antibiotics has been shown to affect immune responses, thus making the host more susceptible to infection (Willing et al., 2011; Pereira et al., 2019; Ma et al., 2023). In addition, many antibiotics exert adversely immunomodulatory effects in birds and mammals (Grondel et al., 1985; Tauber and Nau, 2008; Tykałowski et al., 2013) by directly affecting different immune cell populations, the levels of cytokines synthesized by these cells, and the activity of intracellular enzymes (Chrząstek and Wieliczko, 2015;Terada et al., 2020; Giovagnoni et al., 2023).

The indiscriminate use and misuse of antibiotics are still a sad reality in many countries (Landers et al., 2012). This is particularly the case in countries where there is no legislation regulating the use of antimicrobials in poultry production, and where generic fluoroquinolones and tetracyclines are abundant and inexpensive (Boamah et al., 2016; Da Silva et al., 2023). In these countries, typical management practices include treating newly hatched poults with antibiotics added to drinking water for five consecutive days (Morales-Barrera et al., 2016; Madubuike et al., 2020; Tasmim et al., 2023). In Poland and other counties, fluoroquinolones (in particular enrofloxacin) are used for this purpose due to their broad spectrum of activity, low cost, and pharmacokinetic properties. They eliminate potentially pathogenic bacteria that could infect poults vertically or through contact in the hatchery and during transportation to the farm. Producers use antibiotics in poultry to improve performance, which creates selective pressure conditions conducive to the development and propagation of antimicrobial-resistant microorganisms into the environment, and their further transmission to humans via the food chain may pose a serious threat to public health (Walia et al., 2019; Imperial et al., 2022; Pyzik et al., 2024). The group of antibiotics overused to prevent disease and increase weight gain in poultry includes tetracyclines, and doxycycline ranks first, often being administered on farms without veterinary approval (Tasmim et al., 2023). As measures against the injudicious use of antibiotics in farming, legislative processes were initiated in the European Union, Regulation (EU) 2019/6 of the European Parliament and of the Council of 11 December 2018 on veterinary medicinal products and the metaphylactic-use-limiting legislation of January 2022 (Pyzik et al., 2024) being significant among them. These led to a substantial reduction or complete elimination of the use of selected groups of antibiotics in the poultry sector, but analysis of data in individual European countries has indicated enormous differences in antibiotic application in poultry production. In addition, poultry producers make extensive use of coccidiostats in feed to prevent health problems caused by *Eimeria* spp. parasites. In fact, these drugs not only inhibit or kill pathogens as the desired positive effect, but they also act on the host’s immune system as an undesirable negative effect. Very few studies have investigated the effects of different groups of antimicrobials on the immune system in poultry, and even fewer have focused on turkeys. The present study was conducted to fill this knowledge gap because there is no empirical evidence that prophylactic administration of antibiotics to turkeys in the first days of life does not affect the physiological mechanisms of the immune response after vaccination.

The first aim of this experiment was to determine whether and how enrofloxacin and doxycycline as used in poultry rearing affect the poult immune system function from the time immediately post hatching and during the first weeks of rearing. The second aim was to determine whether there was a related effect from feeding a diet containing the ionophore coccidiostat monensin, which is also commonly administered to farmed birds. Both objectives addressed the research hypothesis that the most commonly used antimicrobial agents on turkey farms can modulate the immune response, especially in response to vaccination. The results have implications for the turkey-rearing industry over much of the world because they raise questions about the effectiveness and cost-effectiveness of metaphylaxis and the welfare of farmed poultry under routine antibiotic regimens.

## Material and methods

### Ethics approval of the study protocol

The protocol for this study was approved by the Local Ethics Committee for Animal Experiments in Olsztyn, Poland (resolution No. 47/2021). All procedures and care related to the animals conformed to the principles found in the EU Directive 2010/63/EU (2010) and to the American Society of Animal Science “Guide for the care and use of agricultural animals in research and teaching, Fourth Edition” (2020).

### Experimental design

The experiment had a two-factorial design. The first factor was treatment, which consisted of four elements: monensin (M), enrofloxacin (E), doxycycline (D), and untreated control (C). The second factor was vaccination schedule, being vaccinated (+) and unvaccinated birds (−) for each treatment group.

### Experimental birds and diet

A total of 3 080 one-day-old Hybrid Converter turkeys (♀) were randomly allocated to eight groups – four groups of unvaccinated birds (*C*−, *M*−, *E*−, and *D*−) and four groups of birds (*C*+, *M*+, *E*+, and *D*+) that were vaccinated. Two feeding phases were planned (Table S1), the first in days of life 1 − 28 and the second in days 29−56, at the end of which the experiment concluded. The birds were fed a different first-phase diet to second-phase diet, and both fulfilled the nutrient requirements of commercial turkeys at their stage of rearing (Hybrid Turkeys, 2020). All birds had unrestricted access to water and feed.

### Housing and biosecurity

Each group consisted of seven replicates with 55 birds per replicate. Each replicate was housed in a 2.5 *m* × 4.0 m pen on litter. Beginning with a replicate of the *C*− group, the adjacent pen held a replicate of the *M*− group, next to it was a replicate of the *E*− group, next to that a replicate of the *D*− group, next to that the second *C*− replicate, the neighboring pen held the second *M*− replicate, and so on. The same scheme was applied for the vaccinated groups of birds. This ensured their uniform distribution in the turkey house. The unvaccinated birds (those from groups *C*−, *M*−, *E*−, and *D*−) were housed in zone No. 1, and the vaccinated birds (those from groups *C*+, *M*+, *E*+, and *D*+) were housed in zone No. 2, the zones being separated by a solid wall. The zones had identical dimensions and equipment, and had separate entrances and hygiene locks for the personnel. The corridors for personnel and feed delivery were independently accessed. Unvaccinated and vaccinated birds were handled by different personnel throughout the experiment, who were required to shower and don disposable protective clothing before entering the zone. Environmental conditions were controlled automatically, adjusted to the birds’ age, established consistently with the recommendations of Hybrid Turkeys (2020), and kept identical for all turkeys.

### Antimicrobials and vaccines

Birds from treatment groups *M*+ and *M*− received the coccidiostat monensin (Coxidin 200, Huvepharma Polska, Warsaw, Poland) at 90 mg/kg of feed for 56 days. Turkeys from treatment groups *E*+ and *E*− received enrofloxacin (Enrofloxacin 10 %, Biowet, Drwalew, Poland), and birds from treatment groups *D*+ and *D*− were administered doxycycline (Doxylin CT WSP 433 mg/g, Dopharma Research B.V., Raamsdonksveer, the Netherlands). Enrofloxacin (10 mg/kg BW, daily) and doxycycline (50 mg/kg BW, daily) were administered with drinking water for the first five days of life. Birds from treatment groups *C*+ and *C*− were the control groups and received no antibiotics or coccidiostats. One-day-old turkeys from groups *C*+, *M*+, *E*+, and *D*+ were administered live-attenuated vaccines against turkey rhinotracheitis (TRT) (Poulvac TRT, Zoetis, Parsippany, NJ, USA) and Newcastle disease (ND) (Nobilis ND clone 30, Merck, Rahway, NJ, USA) by coarse spray, and 28-day-old birds were administered a subcutaneously injected inactivated vaccine against ornithobacteriosis (Ornitin, Phibro, Stamford, CT, USA). Turkeys from groups *C*−, *M*−, *E*−, and *D*− were not vaccinated.

### Sample collection and data acquisition

At 7 and 56 days of age, blood samples were collected from the jugular vein of 7 turkeys per group into sterile tubes containing EDTA-K2 or lithium heparin anticoagulant (for flow cytometry or immunoenzymatic assays, respectively) and into RNAprotect Animal Blood Tubes (Qiagen, Hilden, Germany) for molecular analyses. Then the birds were sacrificed and their spleens were collected. The spleens and blood samples were used for the isolation of mononuclear cells and flow cytometry determination of the percentages of CD4^+^, CD8a^+^, and CD4^+^CD8a^+^ *T* cells within the total T-cell population, and the percentage of IgM^+^ *B* cells within the total B-cell population. For these experiments, 7 birds (each representing the pen average BW ± 10 %) were selected from each treatment group (1 bird per replicate), commonly accepted as the minimum number of turkeys with a unified genotype that ensures reliable, reproducible and statistically significant results without the need for repeating the procedure due to high intra-group variability. During the experiment, the BW of turkeys and feed consumption were recorded pen by pen for 56 days of life to calculate the feed conversion ratio. The mortality rate for the entire rearing period was also calculated.

### Isolation of mononuclear cells and flow cytometry

Mononuclear cells were isolated from the blood and spleens according to a previously described procedure (Koncicki et al., 2012). The cells were counted, and their viability was evaluated using the Vi-Cell XR cell counter (Beckman Coulter, Brea, CA, USA). Viable mononuclear cells (1 × 10^6^) were stained with fluorescein isothiocyanate (FITC)-conjugated Mouse Anti-Chicken CD4 (MCA2164F, Bio-Rad, Kidlington, UK) and phycoerythrin conjugated Mouse Anti-Chicken CD8a (MCA2166PE, Bio-Rad, Kidlington, UK). Separate staining of B cells was carried out with FITC-conjugated Goat Anti-Chicken IgM (AAI27F, Bio-Rad, Kidlington, UK). The percentages of CD4^+^, CD8a^+^, CD4^+^CD8a^+^, and IgM^+^ cells were determined in a FACSCanto II flow cytometer (Becton Dickinson (BD), Franklin Lakes, NJ, USA). Cell staining, acquisition, and analysis of cytometric data were described elsewhere (Kubińska et al., 2014).

### Determination of plasma cytokine levels

The levels of IL-1ß, IL-2, IL-4, IL-6, IL-8, IL-12, IL-13, tumor necrosis factor α (TNF-α), and interferon γ (IFN-γ) were evaluated using commercial ELISA kits according to the protocol provided by the manufacturers (Cat. Nos QY-E80031 (IL-1ß), QY-E80014 (IL-2), QY-E80064 (IL-4), QY-E80013 (IL-6), QY-E80030 (TNF-α), and QY-E80058 (IFN-γ), Qayee Biotechnology, Shanghai, China; Cat. Nos MBS700701 (IL-8), MBS8804359 (IL-12), and MBS031439 (IL-13), MyBioSource, Inc., San Diego, CA, USA). Absorbance was measured with a Sunrise microplate reader (Tecan, Männedorf, Switzerland).

### Analysis of relative IL-6 and IFN-γ gene expression in blood

Genes encoding cytokines that play key roles in regulating immune and inflammatory responses were selected for evaluation of relative gene expression. Total RNA extraction from blood samples was performed using the RNeasy Protect Animal Blood Kit (Qiagen, Hilden, Germany) according to the manufacturer’s instructions. The quantity of the isolated RNA was assessed spectrophotometrically with a Nabi UV/VIS nano-spectrophotometer (MicroDigital, Gyeonggi, South Korea), and its integrity was confirmed through agarose gel electrophoresis (0.8 % concentration). To synthesize complimentary DNA, 1 µg of total RNA underwent reverse transcription using the NG dART RT kit (EURx, Gdańsk, Poland), according to the manufacturer’s protocol. Specific primers (the sequences of which are presented in Table 1) designed for assessing IL-6 and IFN-γ gene expression were developed using Primer 3 software (Whitehead Institute, Cambridge, MA, USA), and synthesized by Genomed (Warsaw, Poland). A real-time PCR was performed on a Quantabio thermocycler (VWR, Radnor, PA, USA) using an SG (SYBR Green) quantitative PCR Master Mix solution (EURx, Gdańsk, Poland). Amplification was conducted according to the following protocol: an initial denaturation step at 95°C for 5 min; 35 cycles with a denaturation phase at 95°C for 10 s, an annealing phase at 53–59°C for 35 s (the temperature dependent on the primer pair used), and an elongation phase at 72°C for 35 s; and a final elongation at 72°C for 5 min. A melting curve analysis was performed over 50–95°C at 0.3°C/s intervals. The relative expression of each gene was calculated using the 2−ΔΔCt method with normalization to efficiency corrections and to the expression levels of the glyceraldehyde-3-phosphate dehydrogenase (GAPDH) and β-actin (ACTB) housekeeping genes.

**Table 1:** PCR primers for amplification of IL-6, IFN-γ, and housekeeping genes in blood samples of 7-day-old poults treated with enrofloxacin or doxycycline antibiotics or a monensin coccidiostat.

Gene	Primer	Sequence (5′–3′)	Melting temperature (°C)	Product size (nucleotides)	GenBank accession number
*IL-6*^1^	Forward	AAGGCGTGGATAGAGAAG	53	158	XM_003207130.4
	Reverse	TGACAGATCGGTAACAGAG			
*IFN-γ*	Forward	AACTGAAGAACTGGACAGAGAGAA	59	160	XM_003202048.3
	Reverse	ACGCCATCAGGAAGGTTGTT			
*ACTB*	Forward	TACCCCATTGAACACGGCAT	58	96	NM_001303173
	Reverse	CTCCTCAGGGGCTACTCTCA			
*GAPDH*	Forward	AGGATACACAGAGGACCAGGTTG	58	71	NM_001303179
	Reverse	CCGCATCAAAGGTGGAGGAATG			

1 Abbreviations: IL-6 = interleukin 6; IFN-γ = interferon gamma; ACTB = β-actin; GAPDH = glyceraldehyde-3-phosphate dehydrogenase.

### Statistical analysis

The results were presented as means and pooled SEM. The data were analyzed by two-way ANOVA with the GLM procedure to examine the main effects of antimicrobials (C, M, E, and D: antibiotic effect (A)), the applied vaccination schedule (+ vs. −: vaccination effect (V)), and their interaction (*A* × *V*). When the model was significant, Tukey’s HSD test was performed to separate treatment means. For performance parameters, a pen was considered as a replicate experimental unit for the statistical analysis. The values of the remaining traits were determined individually in 7 birds per group (1 bird per pen), whose BW was representative of the average BW (± 10 %) in the group. The percentage data of mortality were transformed to arcsines of the square root before analysis. The statistical analysis was performed using Statistica software version 13.1 (TIBCO Software Inc., Palo Alto, CA, USA) at a significance level of *P* < 0.05.

## Results

### Factorial effect on body weight and mortality

Two-way ANOVA for turkey growth performance revealed no significant interactions between the antibiotic factor and the vaccination factor (*A* × *V*). The average BW was greater in 56-day-old turkeys receiving monensin (group M) than in their counterparts from treatment groups C and D. Higher BW was also found in vaccinated turkeys than in unvaccinated birds. Throughout the rearing period, the feed conversion ratio (FCR, kg feed/kg BW) was lower in the group of turkeys receiving monensin than in the other treatments. In general, no significant differences in the mortality rates of turkeys were noted during the 8-week experimental period (Table S2).

### Factorial effect on T- and B-cell subpopulations

Table 2 presents the percentages of T- and B-cell subpopulations in the blood and spleen samples of 7-day-old poults, and Table 3 presents them for the 56-day-old turkeys.

**Table 2:** Percentages of T-cell (CD4^+^, CD8a^+^, and CD4^+^CD8a^+^) and B-cell (IgM)^+^) subpopulations in the blood and spleen of 7-day-old turkeys treated with enrofloxacin or doxycycline antibiotics or a monensin coccidiostat.

Item	n	Blood				Spleen			
		CD4^+^	CD8a^+^	CD4^+^CD8a^+^	IgM^+^	CD4^+^	CD8a^+^	CD4^+^CD8a^+^	IgM^+^
Treatment^1^									
C	14	9.39	2.05	0.63	6.43^a^	43.81	20.96	1.78	7.70
M	14	10.11	1.84	0.71	6.72^a^	45.49	19.71	1.75	7.46
E	14	11.32	1.90	0.47	5.59^b^	44.23	21.07	1.77	7.45
D	14	11.15	1.95	0.53	6.26^ab^	44.01	21.48	1.77	7.20
Vacccination^2^									
−	28	9.41^b^	2.02	0.55	5.89^b^	42.02^b^	21.86^a^	1.84	7.36
+	28	11.58^a^	1.85	0.62	6.61^a^	46.74^a^	19.75^b^	1.69	7.54
Groups									
*C*−	7	8.92	1.93	0.65	6.62^ab^	42.70	20.66	1.74^ab^	7.40
*C*+	7	9.86	2.18	0.60	6.24^abc^	44.91	21.27	1.83^ab^	8.00
*M*−	7	9.04	1.93	0.67	6.11^abc^	43.20	20.96	1.81^ab^	7.43
*M*+	7	11.17	1.76	0.74	7.32^a^	47.77	18.46	1.69^ab^	7.48
*E*−	7	10.03	2.13	0.39	4.99^c^	42.27	22.17	1.76^ab^	7.28
*E*+	7	12.61	1.67	0.56	6.20^abc^	46.19	19.97	1.78^ab^	7.61
*D*−	7	9.64	2.11	0.47	5.83^bc^	39.91	23.67	2.06^a^	7.34
*D*+	7	12.66	1.79	0.59	6.69^ab^	48.10	19.29	1.48^b^	7.05
SEM		0.440	0.090	0.040	0.134	0.670	0.407	0.045	0.143
*P* value									
Antimicrobials (A)		0.342	0.878	0.189	0.006	0.760	0.382	0.993	0.699
Vaccination (V)		0.014	0.344	0.341	0.002	<0.001	0.007	0.091	0.561
*A* × *V* interaction		0.843	0.567	0.793	0.043	0.365	0.143	0.032	0.745

^1^Abbreviations: *C* = untreated control; *M* = treated with monensin (90 mg/kg of feed); *E* = treated with enrofloxacin (10 mg/kg of BW, administered with drinking water for 5 consecutive days after hatching); *D* = treated with doxycycline (50 mg/kg of BW, administered with drinking water for 5 consecutive days after hatching).

^2^Unvaccinated (−) or vaccinated (+) with live-attenuated vaccines against turkey rhinotracheitis and Newcastle disease on day 1.

^a–c^Values in the same column with no common superscripts differ significantly (*P* ≤ 0.05).

**Table 3:** Percentages of T-cell (CD4^+^, CD8a^+^ and CD4^+^CD8a^+^) and B-cell (IgM)^+^) subpopulations in the blood and spleen of 56-day-old turkeys treated with enrofloxacin or doxycycline antibiotics or a monensin coccidiostat.

Item	n	Blood				Spleen			
		CD4^+^	CD8a^+^	CD4^+^CD8a^+^	IgM^+^	CD4^+^	CD8a^+^	CD4^+^CD8a^+^	IgM^+^
Treatment^1^									
C	14	15.92	1.69	0.22	6.23	27.81^ab^	29.11^b^	2.01^b^	11.62^ab^
M	14	15.92	1.81	0.25	6.16	29.01^a^	30.63^ab^	2.18^ab^	11.24^b^
E	14	18.25	1.84	0.22	6.40	24.83^b^	33.90^ab^	2.31^ab^	13.56^ab^
D	14	17.91	1.86	0.23	6.45	28.36^ab^	34.62^a^	2.64^a^	14.61^a^
Vacccination^2^									
**−**	28	15.12^b^	1.74	0.21	6.28	28.41	30.53^b^	1.93^b^	11.18^b^
+	28	18.87^a^	1.86	0.25	6.34	26.59	33.60^a^	2.64^a^	14.33^a^
Groups									
*C*−	7	15.49	1.81	0.23	6.42	28.57	27.40	1.87^b^	11.80^abc^
*C*+	7	16.36	1.58	0.21	6.05	27.06	30.81	2.16^ab^	11.43^bc^
*M*−	7	14.12	1.66	0.23	6.04	30.01	28.44	1.66^b^	10.60^c^
*M*+	7	17.71	1.96	0.28	6.28	28.01	32.81	2.69^a^	11.87^abc^
*E*−	7	15.87	1.79	0.19	6.47	25.09	32.29	1.74^b^	9.88^c^
*E*+	7	20.63	1.89	0.26	6.34	24.57	35.51	2.89^a^	17.24^a^
*D*−	7	15.01	1.70	0.22	6.21	29.99	34.00	2.47^ab^	12.44^abc^
*D*+	7	20.80	2.01	0.24	6.68	26.73	35.24	2.81^a^	16.79^ab^
SEM		0.510	0.079	0.011	0.114	0.538	0.799	0.115	0.540
*P* value									
Antimicrobials (A)		0.106	0.893	0.698	0.803	0.027	0.038	0.009	0.029
Vaccination (V)		<0.001	0.462	0.131	0.819	0.078	0.048	<0.001	0.001
*A* × *V* interaction		0.218	0.626	0.518	0.602	0.813	0.903	0.036	0.017

^1^Abbreviations: *C* = untreated control; *M* = treated with monensin (90 mg/kg of feed for 56 days); *E* = treated with enrofloxacin (10 mg/kg of BW, administered with drinking water for 5 consecutive days after hatching); *D* = treated with doxycycline (50 mg/kg of BW, administered with drinking water for 5 consecutive days after hatching).

^2^Unvaccinated (−) or vaccinated (+) with live-attenuated vaccines against turkey rhinotracheitis and Newcastle disease on day 1, and with inactivated vaccine against *Ornithobacterium rhinotracheale* on day 28.

^a–c^Values in the same column with no common superscript letters differ significantly (*P* ≤ 0.05).

The percentages of CD4^+^ *T* cells in the blood and spleen of 7-day-old birds were significantly affected by vaccination, but not by the antimicrobials administered to birds. The percentages of these cells were greater in vaccinated groups than in unvaccinated groups. The percentages of CD8a^+^ *T* cells were smaller in blood samples from vaccinated turkeys. In the spleen, however, the percentages of these cells were lower in vaccinated groups than in unvaccinated groups. Antimicrobials had no marked effect on the percentages of CD4^+^CD8a^+^ double-positive T cells in the blood or spleen of 7-day-old poults. However, both experimental factors had a notable effect on the IgM^+^ *B* cell subpopulation in the blood of turkeys. The percentages of B cells in blood samples were higher in vaccinated groups than in unvaccinated groups. Neither antimicrobials nor vaccines affected the percentages of this cell subpopulation in the spleen. Enrofloxacin decreased the percentages of these cells in the blood of turkeys, particularly in unvaccinated birds, compared with control group birds and those receiving monensin. Therefore, an interaction effect of antimicrobials and vaccination was noted.

Antimicrobials had no great influence on the percentages of the analyzed T-cell and B-cell subpopulations in the blood of 56-day-old turkeys. The percentages of CD4^+^ *T* cells were higher in the blood of vaccinated groups than in unvaccinated groups. This cell subpopulation in the spleen was affected by the antimicrobials administered to turkeys. The percentages of CD4^+^ *T* cells in the spleen were highest in the groups receiving monensin throughout the rearing period and lowest in birds receiving enrofloxacin with drinking water for the first five days after hatching. Vaccination made the percentages of CD8a^+^ *T* cells higher in the spleens of recipient turkeys than in the spleens of unvaccinated birds. Turkeys administered doxycycline were characterized by higher percentages of these cells in the spleen than control group birds. Vaccination increased the percentages of splenic CD4^+^CD8a^+^ *T*-cell and IgM^+^ *B*-cell subpopulations compared with the percentages in the spleens of unvaccinated birds. A marked interaction effect of antimicrobials and vaccination on the percentages of CD4^+^CD8a^+^ *T* cells and IgM^+^ *B* cells in the spleens of 8-week-old turkeys was also noted.

### Factorial effect on cytokine levels in plasma

The plasma levels of the cytokines analyzed in the samples of the 7-day-old poults are presented in Table 4, and these data for the 56-day-old turkeys are presented in Table 5. Antimicrobials had significant effects on the plasma levels of eight out of the nine cytokines in 7-day-old poults, which varied depending on the antimicrobial substance, cytokine type, and interaction with vaccines. Enrofloxacin lowered the plasma levels of pro-inflammatory cytokines (IL-1β, IL-2, IL-6, TNF-α, and IFN-γ), and raised that of IL-13 (an anti-inflammatory cytokine) in 7-day-old turkeys, relative to the control group. Doxycycline led to a decrease in the plasma levels of IL-6 and TNF-α and increase in plasma IL-8 levels in 7-day-old birds, relative to the control group. Monensin increased the plasma levels of IL-8 and IFN-γ in 7-day-old poults, compared with the control group. Only IL-12 plasma levels were not notably affected by the antimicrobials and coccidiostat administered to these birds. The viruses contained in vaccines administered to turkeys on the first day of life had a significant effect on the plasma levels of eight out of the nine cytokines in 7-day-old birds. The plasma levels of IL-1β, IL-2, IL-12, TNF-α, and IFN-γ were lower in vaccinated 7-day-old poults than in the unvaccinated contingent, whereas the plasma levels of IL-4, IL-13, and IL-8 were higher in vaccinated 7-day-old poults than in their unvaccinated counterparts.

**Table 4:** Levels of cytokines in the plasma of 7-day-old turkeys treated with enrofloxacin or doxycycline antibiotics or a monensin coccidiostat.

Item	n	IL-1ß^1^ (ng/L)	IL-2 (ng/mL)	IL-4 (pg/mL)	IL-6 (pg/mL)	IL-8 (ng/L)	IL-12 (ng/mL)	IL-13 (pg/mL)	TNF-α (ng/mL)	IFN-γ (ng/L)
Treatment										
C	14	23.13^a^	80.91^a^	54.26^ab^	1134.5^a^	64.57^b^	195.8	15.54^b^	587.1^a^	69.64^b^
M	14	24.71^a^	80.21^a^	46.59^b^	1070.8^a^	86.46^a^	177.0	15.44^b^	572.4^a^	84.61^a^
E	14	19.27^b^	58.77^b^	53.83^ab^	835.5^b^	77.02^ab^	169.8	22.63^a^	446.5^b^	69.12^b^
D	14	23.65^a^	74.77^a^	61.13^a^	888.6^b^	86.06^a^	171.5	15.36^b^	393.7^b^	61.46^b^
Vacccination^2^										
−	28	25.62^a^	86.68^a^	41.55^b^	994.7	69.96^b^	232.0^a^	14.65^b^	593.3^a^	83.92^a^
+	28	19.77^b^	60.65^b^	66.35^a^	970.0	87.10^a^	125.0^b^	19.83^a^	406.5^b^	58.49^b^
Groups										
*C*−	7	22.98^ab^	92.51^a^	45.52^bc^	1216.6	51.40^c^	243.9	16.26^b^	684.3	77.70^ab^
*C*+	7	23.28^ab^	69.31^bc^	63.00^abd^	1052.4	77.74^abc^	147.6	14.82^b^	489.8	61.57^bd^
*M*−	7	26.67^a^	87.88^ab^	39.29^c^	1046.8	88.60^ab^	235.9	13.34^b^	665.5	83.26^a^
*M*+	7	22.75^ab^	72.54^bc^	53.88^bc^	1094.8	84.33^ab^	118.1	17.54^b^	479.3	85.96^a^
*E*−	7	26.35^a^	83.51^abc^	40.95^cd^	834.7	73.18^abc^	229.7	14.74^b^	550.0	87.08^a^
*E*+	7	12.20^c^	34.04^d^	66.72^ab^	836.3	80.86^abc^	110.0	30.52^a^	343.0	51.16^cd^
*D*−	7	26.46^a^	82.83^abc^	40.46^c^	880.7	66.65^bc^	218.7	14.26^b^	473.5	87.66^a^
*D*+	7	20.85^b^	66.71^c^	81.80^a^	896.5	105.47^a^	124.3	16.46^b^	313.9	35.26^c^
SEM		0.692	2.714	2.543	41.69	3.128	9.064	0.870	21.63	2.946
*P* value										
Antimicrobials (A)		<0.001	<0.001	0.047	<0.001	0.013	0.351	<0.001	<0.001	<0.001
Vaccination (V)		<0.001	<0.001	<0.001	0.556	0.002	<0.001	<0.001	<0.001	<0.001
*A* × *V* interaction		<0.001	<0.001	0.044	0.285	0.023	0.780	<0.001	0.949	<0.001

^1^Abbreviations: IL = interleukin; TNF-α = tumor necrosis factor α; IFN-γ = interferon γ; *C* = untreated control; *M* = treated with monensin (90 mg/kg of feed); *E* = treated with enrofloxacin (10 mg/kg of BW, administered with drinking water for 5 consecutive days after hatching); *D* = treated with doxycycline (50 mg/kg of BW, administered with drinking water for 5 consecutive days after hatching).

^2^Unvaccinated (−) or vaccinated (+) with live-attenuated vaccines against turkey rhinotracheitis (TRT) and Newcastle disease on day 1.

^a–d^Values in the same column with no common superscripts differ significantly (*P* ≤ 0.05).

**Table 5:** Levels of cytokines in the plasma of 56-day-old turkeys treated with enrofloxacin or doxycycline antibiotics or a monensin coccidiostat.

Item	n	IL-1ß^1^ (ng/L)	IL-2 (ng/mL)	IL-4 (pg/mL)	IL-6 (pg/mL)	IL-8 (ng/L)	IL-12 (ng/mL)	IL-13 (pg/mL)	TNF-α (ng/mL)	IFN-γ (ng/L)
Treatment										
C	14	40.24	81.56	39.68^ab^	1231.2^a^	65.79	243.0^a^	23.59^ab^	594.5^a^	65.80
M	14	40.58	82.84	46.37^a^	1201.3^a^	63.48	232.5^a^	25.79^a^	554.7^ab^	76.81
E	14	34.90	75.31	41.14^ab^	1107.1^ab^	63.84	184.7^b^	24.53^ab^	476.4^b^	77.90
D	14	36.82	80.65	32.55^b^	934.7^b^	57.05	189.1^b^	21.19^b^	469.1^b^	67.52
Vacccination^2^										
−	28	34.96	89.56^a^	36.14^b^	1249.9^a^	63.61	245.4^a^	22.93	590.3^a^	64.17^b^
+	28	41.32	70.62^b^	43.74^a^	987.2^b^	61.48	179.3^b^	24.62	457.0^b^	79.85^a^
Groups										
*C*−	7	37.14	91.54	31.18^bc^	1284.5	64.84	286.1	21.37^b^	624.1	55.13
*C*+	7	43.33	71.58	48.19^ab^	1178.0	66.74	199.9	25.81^ab^	565.0	76.48
*M*−	7	41.40	90.44	36.67^bc^	1374.3	56.54	266.6	22.40^b^	614.4	64.20
*M*+	7	39.77	75.23	56.07^a^	1028.3	70.43	198.4	29.18^a^	494.9	89.41
*E*−	7	33.53	87.34	37.73^bc^	1284.2	66.84	220.6	25.67^ab^	560.4	76.44
*E*+	7	36.27	63.28	44.55^ab^	930.0	60.84	148.9	23.40^ab^	392.4	79.37
*D*−	7	27.75	88.92	38.97^abc^	1056.7	66.22	208.3	22.29^b^	562.3	60.90
*D*+	7	45.90	72.39	26.13^c^	812.7	47.88	169.9	20.09^b^	375.9	74.13
										
SEM		2.218	2.308	1.803	38.29	2.206	7.582	0.592	16.98	2.498
*P* value										
Antimicrobials (A)		0.772	0.566	0.014	0.007	0.509	<0.001	0.015	0.002	0.128
Vaccination (V)		0.163	<0.001	0.011	<0.001	0.622	<0.001	0.094	<0.001	0.001
*A* × *V* interaction		0.451	0.865	0.001	0.477	0.071	0.445	0.002	0.315	0.304

^1^Abbreviations: IL = interleukin; TNF-α = tumor necrosis factor alpha; IFN-γ = interferon γ; *C* = untreated control; *M* = treated with monensin (90 mg/kg of feed, for 56 days); *E* = treated with enrofloxacin (10 mg/kg of BW, administered with drinking water for 5 consecutive days after hatching); D – treated with doxycycline (50 mg/kg of BW, administered with drinking water for 5 consecutive days after hatching).

^2^Unvaccinated (−) or vaccinated (+) with live-attenuated vaccines against turkey rhinotracheitis and Newcastle disease on day 1, and with inactivated vaccine against *Ornithobacterium rhinotracheale* on day 28.

^a–c^Values in the same column with no common superscripts differ significantly (*P* ≤ 0.05).

Monensin had no noteworthy influence on the plasma levels of the analyzed cytokines in 56-day-old turkeys. Enrofloxacin decreased the plasma levels of IL-12 and TNF-α. Birds receiving doxycycline were characterized by lower plasma levels of IL-6, IL-12, and TNF-α at 56 days of life than control-group birds. Interleukin-2, IL-6, IL-12, and TNF-α were less concentrated in the plasma of vaccinated turkeys at the experiment’s end, whereas IL-4 and IFN-γ were more concentrated in the plasma of these birds, compared with their unvaccinated counterparts’ samples. Neither antimicrobials nor vaccines affected the plasma levels of IL-1β and IL-8 in 56-day-old turkeys.

### Factorial effect on relative expression of cytokine genes in plasma

Table 6 sets out the data for relative expression of the *IL-6* and *IFN-γ* genes in blood sampled from 7-day-old poults and 56-day-old turkeys. Expression of the *IL-6* gene was significantly downregulated in the blood of vaccinated 56-day-old turkeys. A downregulation of *IL-6* gene expression was also noted in the blood of 7-day-old birds under the influence of enrofloxacin, compared with the control groups. Doxycycline also induced a moderate reduction in *IL-6* gene expression in the blood of turkeys at both ages. Although antimicrobials affected plasma IFN-γ levels in 7-day-old poults, and vaccines affected the plasma levels of this cytokine in 56-day-old turkeys, *IFN*-*γ* gene expression in the blood of turkeys did not differ significantly between groups irrespective of bird age.

**Table 6:** Relative expression of genes encoding interleukin 6 (IL-6) and interferon γ (IFN-γ) in the blood of 7- and 56-day-old turkeys treated with enrofloxacin or doxycycline antibiotics or a monensin coccidiostat.

Item	n	IL-6		IFN-γ	
		7 d	56 d	7 d	56 d
Treatment^1^					
C	14	0.27^a^	0.29	0.95	0.97
M	14	0.27^a^	0.29	0.97	0.98
E	14	0.23^b^	0.28	0.95	0.98
D	14	0.25^ab^	0.26	0.94	0.98
Vacccination^2^					
−	28	0.26	0.29^a^	0.97	0.97
+	28	0.26	0.27^b^	0.94	0.99
Groups					
*C*−	7	0.28	0.29	0.95	0.95
*C*+	7	0.27	0.28	0.95	0.98
*M*−	7	0.27	0.30	0.97	0.97
*M*+	7	0.27	0.28	0.98	0.99
*E*−	7	0.23	0.29	0.97	0.98
*E*+	7	0.23	0.26	0.94	0.99
*D*−	7	0.25	0.28	0.98	0.97
*D*+	7	0.25	0.25	0.90	0.98
					
SEM		0.004	0.004	0.008	0.007
*P* value					
Antimicrobials (A)		<0.001	0.072	0.494	0.800
Vaccination (V)		0.961	0.002	0.075	0.196
*A* × *V* interaction		0.940	0.724	0.217	0.886

^1^Abbreviations: *C* = untreated control; *M* = treated with monensin (90 mg/kg of feed, for 56 days); *E* = treated with enrofloxacin (10 mg/kg of BW, administered with drinking water for 5 consecutive days after hatching); *D* = treated with doxycycline (50 mg/kg of BW, administered with drinking water for 5 consecutive days after hatching).

^2^Unvaccinated (−) or vaccinated (+) with live-attenuated vaccines against turkey rhinotracheitis and Newcastle disease on day 1, and with inactivated vaccine against *Ornithobacterium rhinotracheale* on day 28.

^a and b^Values in the same column with no common superscripts differ significantly (*P* ≤ 0.05).

## Discussion

Antimicrobials are widely used on commercial poultry farms to treat and prevent bacterial and parasitic diseases (Landers et al., 2012; Singer et al., 2023a, 2023b). In developed countries, where emphasis has been placed on educating veterinarians and poultry producers and where national programs have been implemented to reduce antimicrobial use and the need for antimicrobial therapies, veterinary antimicrobial consumption (mg/kg BW) has decreased year on year (Singer et al., 2023a, 2023b). In contrast, antimicrobial use in Brazil, China, India, Russia, and South Africa is expected to increase by 99 % by 2030 relative to 2010 data (Van Boeckel et al., 2015), most likely due to the growing number of birds raised in these countries. Śmiałek et al. (2023) reported that antibiotic consumption (mg/kg BW) in broiler turkey flocks in Poland decreased by an average of 42.25 % between 2019 and 2021.

While antibiotics are increasingly restricted and decreasingly used in the developed world, ionophore coccidiostats remain widely permitted and utilized as an alternative means of maintaining flock health in the same geographical region. The addition of coccidiostats to feed during most of the rearing period is the cheapest and most common method of preventing coccidiosis in broiler chickens and turkeys (Martins et al., 2022). The monensin used in the present study is an ionophore antibiotic and the most popular coccidiostat on turkey farms in Poland, and also one of the most commonly applied coccidiostats in the world (Chapman, 2008). Mikulski et al. (2022) demonstrated that monensin administered to turkeys in the diet up to the 12^th^ week had a beneficial effect on BWG (kg/bird) and FCR (kg feed/kg BWG). Also in our experiment, 56-day-old turkeys which had received monensin in feed had higher BW and lower FCR than birds which had not. Ionophore antibiotics have antimicrobial activity mediated by their ability to exchange Na^+^ and *K*^+^ ions across the cell membrane, thereby disrupting ionic gradients and altering cellular physiology. In addition to veterinary applications, monensin exhibits a broad spectrum of activity against opportunistic human pathogens, including bacteria, viruses, fungi, and parasites (Rajendran et al., 2018). It also exerts anti-inflammatory and immunomodulatory effects (Allam et al., 2024). One way in which it does so is by inducing the apoptosis of mast cells and eosinophils through a secretory granule–mediated pathway (Maccarana et al., 2022; Vraila et al., 2023). In the current study, monensin led to an appreciable increase in plasma IL-8 levels, which was associated with an increase in IFN-γ levels, in 7-day-old poults vaccinated against ND and TRT. Interleukin-8 plays a critical role in various biological processes, including the recruitment of immune cells to inflammation sites and the regulation of immune cell activity. However, in 56-day-old turkeys, monensin had no notable effect on the levels of the analyzed cytokines or the percentages of the analyzed T-cell and B-cell subpopulations. Therefore, the effect of this coccidiostat on the plasma levels of IFN-γ and IL-8 depended on the effect exerted by the viruses contained in vaccines administered to one-day-old poults. Abdelhady et al. (2021) described the anti-inflammatory activity of monensin, which decreased *IL-6* and *IFN-γ* gene expression in the jejunal tissues of 35-day-old broiler chickens infected with mixed *Eimeria* species. Monensin is believed to be one of the safest coccidiostats for poultry, including turkeys, which are particularly sensitive to some ionophore antibiotics (Chapman, 2008). However, our previous study revealed that monensin induced significant morphological changes in the tissues of central and peripheral lymphoid organs in turkeys fed diets containing it, compared with the same tissues of control birds (Smagieł et al., 2023). Turkeys receiving monensin may, therefore, be characterized by lower vaccine-induced antibody titers than birds fed diets without the coccidiostat (Ognik et al., 2024).

The intestinal tract harbors a diverse community of microbes that have co-evolved with the host immune system. The communication between microbiota and the immune system is mediated by the interaction of bacterial components with pattern-recognition receptors on intestinal epithelium and various antigen-presenting cells. These interactions activate both innate and adaptive immune responses. Interaction between the microbial community and host plays a crucial role in the mucosal homeostasis and health status of the host. Antibiotics can impair host immunity by disrupting the gut microbiome. This disruption can lead to a decrease in microbial diversity and changes in the types of bacteria present, impacting colonization resistance and making the host more susceptible to infections. The gut microbiota produces various metabolites, such as short-chain fatty acids, which stimulate the production of cytokines. The physiological microbiota influences the priming signal of the inflammasome activation that leads to the transcription of TNF-α and IL-6, as well as pro-IL-1β and pro-IL-18 (Kogut et al., 2020). Antibiotic-induced dysbiosis can alter the production of these metabolites, affecting immune function. Previous research by Mikulski et al. (2022) demonstrated that treating poults with enrofloxacin or doxycycline in the first days post hatching induced a long-term decline in selected cecal enzyme activity. They also noted elevated noradrenaline levels in 8-week-old turkeys as effects of the earlier administration of these antibiotics or provision of feed containing monensin. Lower serotonin and higher cortisol plasma levels were also observed with doxycycline administration. This may indicate changes in gut microbiota activity to be the most probable cause of the stress induction in the treated turkeys, given that this microbiota is a major functional element of the gut–brain axis. Gut bacteria release endotoxins, which are among the metabolites which may potentially affect the expression levels of neurotransmitters as well as their precursors and receptors in the central nervous system through blood flow and vagus-dependent pathways, thus influencing brain function (Farzi et al., 2018).

Antimicrobials not only act on microorganisms, but also significantly affect the host and exhibit non-antibiotic activity. Almost all antibiotic classes exert effects on the immune response. Sulfonamides, tetracyclines, β-lactams, aminoglycosides, lincosamides, cephalosporins, macrolides, and fluoroquinolones either increase or decrease phagocyte functions (Labro, 2000; Van der Meer, 2003; Yudhawati and Wicaksono, 2024). The underlying mechanisms of immunomodulation of many of these agents have not been fully elucidated. What is known is that the immunomodulatory and anti-inflammatory effects exerted by some antibiotics significantly attenuate the immune response to a vaccine, thus reducing protection (Shen et al., 2024).

Many of the above antibiotics are administered to birds in the first days of life, when the immune system matures, the gut microbiome develops, and vaccination programs are implemented. Administering antibiotics to chicks is common practice in many countries, where it is a part of metaphylaxis in the broad sense, i.e. taking measures to negate the effects of cyclic infections on a particular farm. It should be emphasized that there are enormous differences between individual European countries in terms of procedures for managing poultry production, the choice and availability of specific antimicrobials, vaccine availability, and the individual approach of veterinarians and poultry farmers (Pyzik et al., 2024).

The doxycycline and enrofloxacin administered to turkeys in this experiment are often used in poultry production. They have a wide range of non-antibiotic properties (Navarro-Triviño et al., 2020; Assar et al., 2021; Ma et al., 2023). The available literature indicates that antibiotics can induce the production of free radicals, which is related to their mechanism of action. Enrofloxacin can generate reactive oxygen species that can damage DNA, proteins, and lipids, leading to bacterial cell death and also to deterioration of host cell functioning (Badawy et al., 2021). Doxycycline also exerts oxidative action, and one stronger than monensin’s. However, the effects of doxycycline are highly dose dependent. While sufficiently high doses of this antibiotic may result in increased production of free radicals, probably resulting from its interference with cellular energy metabolism (Tan et al., 2017), pharmacologically appropriate doses of doxycycline actually reduce oxidative stress, thereby inhibiting lipid peroxidation and inflammatory reactions (Clemens et al., 2018). Previous research by Smagieł et al. (2024) demonstrated that both enrofloxacin and doxycycline induced oxidative stress in poults when administered directly after hatching. Enrofloxacin exerted this effect for a markedly longer period – it persisted even up to 7 weeks after treatment. Doxycycline acted for a much shorter period and actually stimulated antioxidative mechanisms after declining to low levels in the turkeys. Monensin, in turn, did not influence the oxidative stress markers measured conspicuously. Oxidative stress plays a crucial role in modulating various cell signaling pathways, including mitogen-activated protein kinases, nuclear factor kappa B (NF-κB), nuclear factor erythroid 2-related factor 2, and phosphoinositide 3-kinase/protein kinase B. Many distinct genes’ expression has been linked to the activation of these transcription factors (Oke et al., 2024). It has long been believed that NF-κB is one of the key factors in cellular signaling that activates the immune system in response to a variety of stimuli, oxidative stress among them. The mechanisms by which antibiotics, and especially quinolones affect various cytokines and chemokines include the regulation of key transcription factors such as NF-κB (Ghosh and Karin, 2002; Dalhoff and Shalit, 2003).

The enrofloxacin used in this study exerted a stronger immunomodulatory effect than the other two antimicrobials, and it decreased the plasma levels of the IL-1β, IL-2, IL-6, IL-12, and TNF-α proinflammatory cytokines in 7-day-old poults. The decrease in the plasma levels of IL-1β, IL-2, and IL-12 was more pronounced in turkeys vaccinated against ND and TRT during enrofloxacin treatment. In addition, a significant decrease in relative IL-6 gene expression was noted in the blood of these birds. A similar phenomenon was observed previously and stated to depend on and be regulated by IL-1β and TNF-α levels (Cahill and Rogers, 2008; Chen et al., 2016). Inhibition of the synthesis of cytokines (IL-2, IL-12, and TNF-α) involved in the cell-mediated immune response (type 1 T helper; Th1) led to a significant increase in the plasma levels of IL-4 and IL-13 in the present cohort of 7-day-old poults vaccinated on the first day of life with preparations containing live-attenuated viruses. There is evidence to suggest that chicken IL-4 functions similarly to its mammalian analog by enhancing humoral – type 2 T helper (Th2) – immunity. However, Williams et al. (2005) demonstrated that quinolones (ciprofloxacin and moxifloxacin) inhibited the secretion of IL-4 and IFN-γ by CD3^+^CD4^+^ cells. The ability of pathogens to stimulate preferentially either a Th1 or Th2 response depends on several factors. When antibacterial agents inhibit cytokine generation with variable effects on immune response, they may alter the response induced by vaccination (Williams et al., 2005; Shen et al., 2024).

In the present study, enrofloxacin also induced a significant decrease in the percentage of IgM^+^ *B* cells in the blood of 7-day-old poults, particularly in the unvaccinated subgroup. Similar observations were made by Chrząstek et al. (2011) and Chrząstek and Wieliczko (2015), who reported a highly significant rise in the percentages of B cells in the bursae of Fabricius and spleens of chickens receiving enrofloxacin for the first five days after hatching. It appears that the fall in the percentage of B cells observed in this study was associated with low plasma IL-2 levels. This cytokine is essential for the immune response, is a powerful growth factor for a variety of cell types, and plays a key role in T-cell differentiation and B-cell development (Miyamoto et al., 2001). Quinolones, in general, exert their modulatory effects when combined with a co-stimulant (Yudhawati and Wicaksono, 2024). It should be emphasized that the modulatory effects of quinolones are not observed when they are used alone, and an extra stimulatory effect is required. In the current study, the viruses contained in the vaccines administered to birds during enrofloxacin treatment acted as co-stimulants. Therefore, a significant interaction effect of antibiotics (especially enrofloxacin) and vaccines was observed on many of the analyzed immunological indicators in both 7- and 56-day-old turkeys. These observations corroborate the results of our previous study investigating the effect of enrofloxacin on the immune system of young, clinically healthy, unvaccinated broiler chickens (Jankowski et al., 2022). Enrofloxacin administered to birds for the first five days after hatching led to a significant elevation of IL-2 levels and the percentage of B cells (CD3^-^Bu-1^+^) in the blood of 6-day-old chicks.

When administered with drinking water for the first five days after hatching, doxycycline induced the plasma levels of pro-inflammatory cytokines such as IL-6 (a central player in the regulation of host defense mechanisms), IL-12, and TNF-α to be substantially lower not only in 7-day-old poults but also in the turkeys when they had reached the 56^th^ day of life. These findings are quite similar to the results of a study conducted by Feng et al. (2021) on broiler chickens infected with the avian infectious bronchitis virus. They found that doxycycline exerted anti-inflammatory effects by decreasing the relative expression of genes encoding cytokines (*IL-1β, IL-6, TNF-α*, and *IFN-γ*) in the trachea and lungs of infectious bronchitis virus–infected chickens. Interleukin 6 is produced early after infection as part of the induced innate immune response. Together with IL-1 and TNF-α, IL-6 stimulates the acute phase response (Schneider et al., 2001; Cray et al., 2009). Our previous study revealed that doxycycline administered to turkeys in the first days of life significantly decreased serum anti-ND virus antibody titers (Ognik et al., 2024). The effects of tetracyclines manifest primarily in the inhibition of phagocytic functions. These antibiotics reduce the synthesis of oxides of nitrogen (NO_X_) and reactive free radicals in granulocytes (Labro, 2000). Smagieł et al. (2024) observed the inhibitory effect of doxycycline, enrofloxacin, and monensin on inducible nitric oxide synthase (iNOS) gene expression in the blood of poults compared with the control group. The effect was associated with reduced blood NO levels in 7-day-old birds. In addition, doxycycline significantly decreased the serum levels of NO_X_, which is responsible for the synthesis of reactive oxygen species and the development of the inflammatory response. In the present study, doxycycline had no effect on the proportions of the analyzed T-cell or B-cell subpopulations in the blood of turkeys. However, in the spleens of 56-day-old turkeys, doxycycline increased the percentages of CD8^+^ and CD4^+^CD8^+^ *T* cells, particularly in birds vaccinated against ornithobacteriosis with an inactivated-bacteria preparation. It is difficult to compare these results with previous findings, because similar experiments have not been performed on turkeys or other poultry species. In the work of Pomorska-Mól et al. (2014) on pigs (where IL-6 functions similarly to in avian species), the animals were first administered doxycycline for five days, and then were given prophylaxis against pseudorabies with a live-attenuated vaccine; two weeks after the last vaccination, the percentage of CD4^+^CD8^+^ *T* cells in their blood had decreased significantly, which indicates an opposite effect to that noted in turkeys in the current experiment.

## Conclusions

The study demonstrated that antimicrobials administered to turkeys exert various non-antibiotic effects on the host. They affect the levels of key cytokines responsible for polarizing the immune response toward the humoral or cellular, and interfere with the interaction between the immune system and the pathogens contained in vaccines by modulating the immune response. There may appear to be commercial advantage in administering antibiotics to clinically healthy poults, although medicating hatchling turkeys in this way is often irrational and therapeutically unjustified; however, these findings should be considered when indiscriminate antibiotic provision is contemplated, as such treatment may result in the absence of the expected vaccine-induced protection and increased susceptibility of the birds to environmental pathogens. Therefore, antibiotics should only be administered in the early post-hatching period in confirmed cases of diseases caused by microorganisms sensitive to these drugs, when the expected health benefits outweigh the risk of impaired immunity. These complex trade-offs between therapeutic benefits and immune consequences are implicated in the challenges of modern intensive poultry production, and their consideration by industry operators is essential.

## CRediT authorship contribution statement

**B. Tykałowski:** Writing – original draft, Resources, Methodology, Formal analysis, Conceptualization. **J. Kowalczyk:** Resources, Methodology, Formal analysis. **K. Ognik:** Writing – original draft, Resources, Project administration, Methodology, Formal analysis, Conceptualization. **D. Mikulski:** Resources, Methodology, Investigation, Conceptualization. **A. Koncicki:** Writing – review & editing, Supervision, Formal analysis, Conceptualization. **K. Kozłowski:** Writing – review & editing, Supervision, Project administration, Methodology, Conceptualization. **J. Jankowski:** Writing – review & editing, Supervision, Project administration, Methodology, Funding acquisition, Formal analysis, Conceptualization.

## Disclosures

No conflict of interest exists in the submission of this manuscript, and manuscript is approved by all authors for publication. On behalf of all the authors, I declare that this paper is original and none of the material in the paper has been published or is under consideration for publication elsewhere. All the authors listed have read the manuscript and approved the submission of the paper to this journal.

## Appendix Supplementary materials

Image, application 1

Image, application 2

## Footnotes

Supplementary material associated with this article can be found, in the online version, at doi:10.1016/j.psj.2025.105782.
