# Supplementary material for: Early post-hatching effects of antibiotics and ionophore coccidiostat supplementation on immune parameters in turkeys
Source: Poult Sci. 2025 Sep 4;104(11):105782. doi: 10.1016/j.psj.2025.105782 (PMC12683119; doi:10.1016/j.psj.2025.105782)
Supplement: Supplementary file 1 [file mmc1.docx]

**Table S1. Ingredient composition and nutrient content of diets fed to turkeys treated with enrofloxacin or doxycycline antibiotics or a monensin coccidiostat (g/100 g, as-fed basis)**

| Item | Feeding period, days of life | |
| --- | --- | --- |
|  | 0–28 | 29–56 |
| Ingredients |  |  |
| Wheat | 26.280 | 41.666 |
| Maize | 20.000 | 10.000 |
| Soybean meal (48% CP) | 42.690 | 34.736 |
| Rapeseed meal | 3.000 | 4.000 |
| Soybean oil | 3.073 | 5.083 |
| Sodium bicarbonate | 0.200 | 0.200 |
| Sodium chloride | 0.152 | 0.160 |
| Limestone | 1.399 | 1.413 |
| Monocalcium phosphate | 2.096 | 1.696 |
| L-lysine HCl | 0.397 | 0.416 |
| DL-methionine | 0.291 | 0.227 |
| L-threonine | 0.072 | 0.053 |
| Choline chloride | 0.100 | 0.100 |
| Vitamin-mineral premix^[1](https://www.sciencedirect.com/science/article/pii/S0032579123003954?via%3Dihub#tb1fn1)^ | 0.250 | 0.250 |
| Calculated nutrient content |  |  |
| Metabolizable energy, kcal/kg | 2800 | 2950 |
| Crude protein | 27.00 | 24.50 |
| Lysine total | 1.75 | 1.58 |
| Methionine total | 0.67 | 0.58 |
| Methionine + Cys total | 1.12 | 1.00 |
| Threonine total | 1.08 | 0.95 |
| Calcium | 1.20 | 1.10 |
| Available phosphorus | 0.58 | 0.50 |
| Na | 0.14 | 0.14 |
| Analyzed chemical composition |  |  |
| Crude protein | 27.14 | 24.09 |
| Crude fat | 3.47 | 7.07 |

^1^ Provided per kg diet (feeding periods: days of life 0 to 28 and 29 to 56) in mg: retinol 3.78 and 3.38, cholecalciferol 0.13 and 0.12, α-tocopheryl acetate 100 and 90, vit. K_3_ 5.8 and 5.6, thiamine 5.4 and 4.7, riboﬂavin 8.4 and 7.5, pyridoxine 6.4 and 5.6, cobalamin 0.032 and 0.028, biotin 0.32 and 0.28, pantothenic acid 28 and 24, nicotinic acid 84 and 75, folic acid 3.2 and 2.8, Fe 64 and 60, Mn 120 and 112, Zn 110 and 103, Cu 23 and 19, I 3.2 and 2.8, Se 0.30 and 0.28, respectively.
