# Supplementary material for: Early post-hatching effects of antibiotics and ionophore coccidiostat supplementation on immune parameters in turkeys
Source: Poult Sci. 2025 Sep 4;104(11):105782. doi: 10.1016/j.psj.2025.105782 (PMC12683119; doi:10.1016/j.psj.2025.105782)
Supplement: Supplementary file 2 [file mmc2.docx]

**Table S2. Production parameters on the 56^th^ day of life for turkeys treated with enrofloxacin or doxycycline antibiotics or a monensin coccidiostat**

| Item | BW (kg) | FCR^1^ (kg/kg) | Mortality (%) |
| --- | --- | --- | --- |
| Treatment |  |  |  |
| C | 4.63^b^ | 1.75^a^ | 2.99 |
| M | 4.74^a^ | 1.70^b^ | 3.90 |
| E | 4.65^ab^ | 1.76^a^ | 2.99 |
| D | 4.61^b^ | 1.77^a^ | 4.94 |
| Vacccination^2^ |  |  |  |
| − | 4.78^a^ | 1.75 | 4.29 |
| + | 4.53^b^ | 1.74 | 3.12 |
| Group |  |  |  |
| C − | 4.75 | 1.75 | 2.86 |
| C + | 4.52 | 1.75 | 3.12 |
| M − | 4.87 | 1.70 | 3.90 |
| M + | 4.61 | 1.71 | 3.90 |
| E − | 4.80 | 1.76 | 3.38 |
| E + | 4.50 | 1.76 | 2.60 |
| D − | 4.73 | 1.79 | 7.01 |
| D + | 4.48 | 1.74 | 2.86 |
| SEM | 0.022 | 0.006 | 0.507 |
| P-value |  |  |  |
| Antimicrobials (A) | 0.006 | <0.001 | 0.784 |
| Vaccination (V) | <0.001 | 0.248 | 0.232 |
| A × V interaction | 0.842 | 0.148 | 0.358 |

^1^Abbreviations: FCR = feed conversion ratio (from day 0 to day 56); C = untreated control; M = treated with monensin (90 mg/kg of feed, for 56 days); E = treated with enrofloxacin (10 mg/kg of BW, administered with drinking water for 5 consecutive days after hatching); D = treated with doxycycline (50 mg/kg of BW, administered with drinking water for 5 consecutive days after hatching)

^2^ Unvaccinated (−) or vaccinated (+) with live-attenuated vaccines against turkey rhinotracheitis and Newcastle disease on day 1, and with inactivated vaccine against *Ornithobacterium rhinotracheale* on day 28.

Data represent mean values of 7 pens (replicates) per group.

^a and b^ Values in the same column with no common superscripts differ significantly (*P* ≤ 0.05).
